# Supplementary figures and images for: Copper Amine Oxidase (CuAO)-Mediated Polyamine Catabolism Plays Potential Roles in Sweet Cherry (Prunus avium L.) Fruit Development and Ripening
Source: Int J Mol Sci. 2022 Oct 11;23(20):12112. doi: 10.3390/ijms232012112 (PMC9603101; doi:10.3390/ijms232012112)

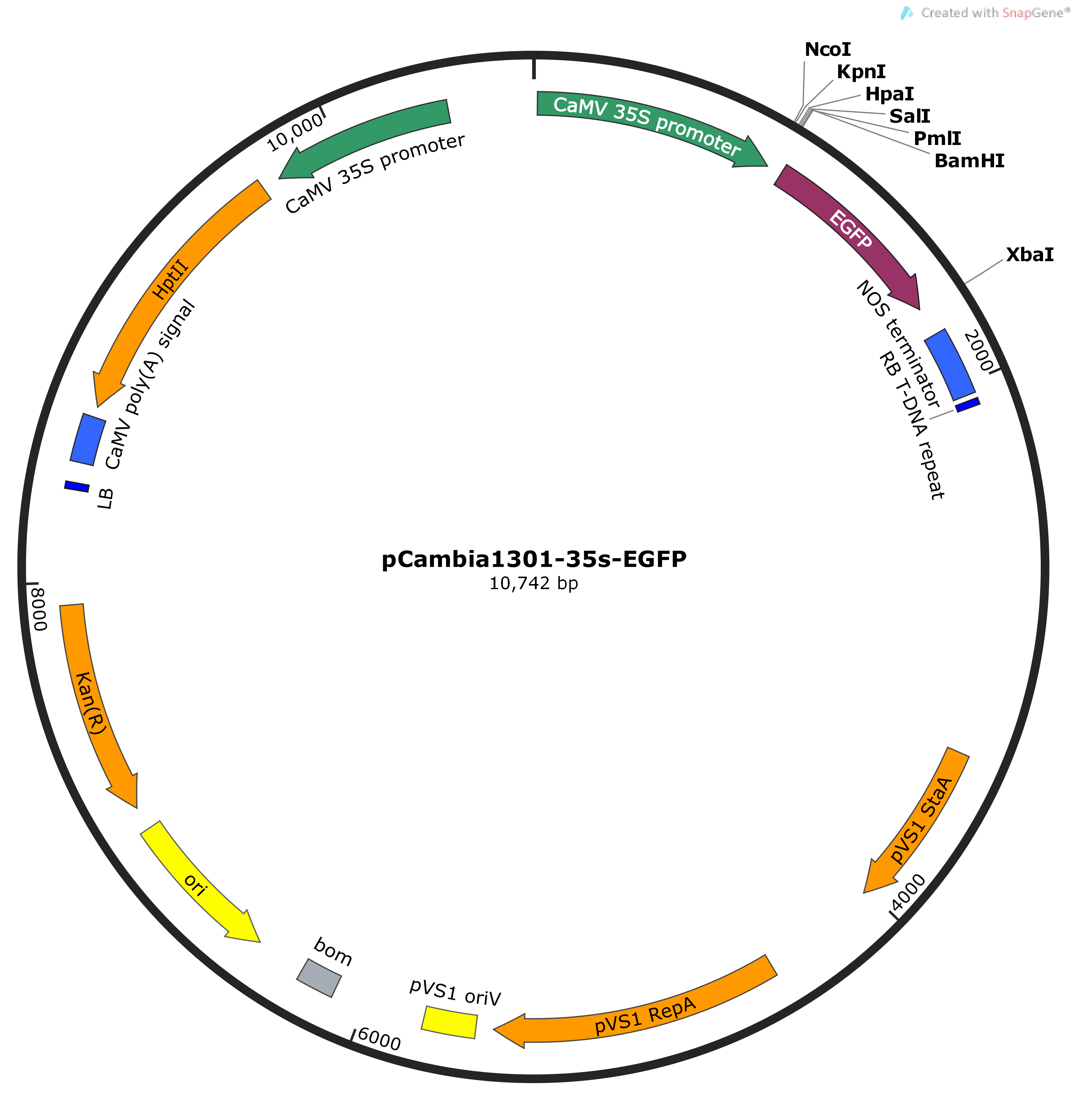

Supplement: Supplementary file 1 [file ijms-23-12112-s001.zip › supplementary files/Figure S1. pCambia1301-35s-GFP vector Map.tiff]
